# Psychometric study of the SF-36v2 in hereditary angioedema due to C1 inhibitor deficiency (C1-INH-HAE)

**DOI:** 10.1186/s13023-022-02202-2

**Published:** 2022-03-02

**Authors:** Paola Palao-Ocharan, Nieves Prior, Elia Pérez-Fernández, Magdalena Caminoa, DV-HAE-QoL Study Group, W. Aberer, S. Betschel, A. Bygum, R. A. Campos, D. Csuka, H. Farkas, C. Gómez-Traseira, A. S. Grumach, I. Leibovich, A. Malbran, D. Moldovan, E. Mihaly, K. Obtulowicz, G. Porebski, A. Reshef, P. Staubach, Teresa Caballero

**Affiliations:** 1 Allergy Unit, Hospital Regional Universitario de Málaga, Málaga, Spain; 2 Allergy Department, Hospital Universitario Severo Ochoa, Leganés, Madrid, Spain; 3 Research Unit, Hospital Universitario Fundación Alcorcón, Madrid, Spain; 4 Clínica Marazuela, Talavera de La Reina, Toledo, Spain; 5 Allergy Department, Hospital Universitario La Paz, Madrid, Spain; 6 Hospital La Paz Institute for Health Research (IdiPaz), Madrid, Spain; 7 Biomedical Research Network On Rare Diseases (CIBERER, U754), Madrid, Spain; 8 Department of Dermatology, Medical University, Graz, Austria; 9 Division of Allergy and Clinical Immunology, St. Michael’s Hospital, Toronto, Canada; 10 Department of Clinical Genetics, Odense University Hospital, Odense, Denmark; 11 Clinical Institute, University of Southern Denmark, Odense, Denmark; 12 Departamento de Medicina Interna E Suporte Diagnostico, Faculta de Medicina da Bahia, Salvador, BA Brazil; 13 Hungarian Angioedema Reference Center, 3Rd Department of Internal Medicine, Semmelweis University, Budapest, Hungary; 14 Clinical Immunology, Medical School, University Center Health ABC, Santo André, São Paulo, Brazil; 15 Allergy, Immunology, and Angioedema Center, Barzilai University Medical Center, Ashkelon, Israel; 16 Allergy, Asthma and Immunology Unit, Buenos Aires, Argentina; 17 Department of Allergology–Immunology, Mures County Hospital, Tìrgu-Mures, Romania; 18 Department of Clinical and Environmental Allergology, Jagiellonian University, Krakow, Poland; 19 Department of Dermatology, University Medical Center Mainz, Mainz, Germany

**Keywords:** Quality of life, Hereditary angioedema, C1-inhibitor, Questionnaire, Psychometric study, SF-36v2, HAE-QoL

## Abstract

**Background:**

The generic 36-item Short-Form Health Survey (SF-36v2) has been used to assess health related quality of life in adult patients with hereditary angioedema due to C1-inhibitor deficiency (C1-INH-HAE) even though it has not yet been validated for use in this specific disease.

**Objective:**

This study aims to validate the SF-36v2 for use in adult patients with C1-INH-HAE.

**Results:**

There was a very low item non-response rate (1–3.4%), with a high ceiling effect in 25/35 items and a low floor effect in 3/35 items. A moderate ceiling effect was observed in 5/8 dimensions of the SF-36v2, whereas no floor effect was noticed in any of the dimensions. Internal consistency was good to excellent with Cronbach's alpha coefficient ranging between 0.82 and 0.93 for the different dimensions. Construct validity was good: seven out of the 8 hypotheses defined on clinical criteria were confirmed, discriminant validity assessment showed significant differences among patients with different C1-INH-HAE severity, convergent validity showed a good correlation among the physical and mental component summaries of the SF-36v2 and the HAE-QoL total score (0.45 and 0.64 respectively, *P* < 0.001). Test–retest reliability was high with intraclass correlation coefficient varying from 0.758 to 0.962. The minimal clinically important difference was calculated by distribution methods and small differences in the domain scores and in the component summaries scores were shown to be meaningful.

**Conclusions:**

The psychometric properties of the SF-36v2 show it can be a useful tool to assess HRQoL in adult patients with C1-INH-HAE, although with some content validity limitation.

**Methods:**

The psychometric properties of the SF-36v2 were evaluated in an international setting based on responses from 290 adult C1-INH-HAE patients in 11 countries.

## Introduction

Hereditary Angioedema due to C1 inhibitor deficiency (C1-INH-HAE) is a rare disease characterized by recurrent episodes of subcutaneous and/or submucosal edema, that may cause significant morbidity and be life-threatening [1, 2]. A recent systematic review of epidemiologic studies estimates that its prevalence varies between 1.1 and 1.6 per 100,000 inhabitants [3]. The minimal prevalence rate in Spain was 1.09:100,000 inhabitants in 2003 [4].

C1-INH-HAE may cause significant morbidity due to the unpredictability of angioedema attacks, painful attacks, risk of asphyxia and the need for emergency intervention [5]. Different factors such as the low prevalence of the disease, hereditary transmission, improper diagnosis, concern about transmission of the disease to children, worry about access to specific treatments, and side effects of treatments, among others, have a negative effect on health-related quality of life (HRQoL) [6–11].

There are currently no reliable biomarkers to monitor the activity of C1-INH-HAE during follow-up visits. HRQoL measurement tools have been recommended for use in clinical practice to facilitate communication, modify or establish therapeutic action, discuss patients’ hidden problems, and monitor treatment response [1, 12]. The SF-36v2 (SF-36 from now on) is a generic health survey that can be applied both to the general population as well as to specific conditions [13]. This tool has demonstrated good psychometric properties that have been assessed in over 400 articles [14], however its psychometric characteristics in this disease are unknown. It is of special interest to have a generic HRQoL assessment scale validated for C1-INH-HAE. This will lead to a better interpretation of the HRQoL results for patients with C1-INH-HAE who complete SF-36 and allow for comparisons across disease groups.

The main aim of this study was to assess the psychometric properties of the generic SF-36 questionnaire in adult patients with C1-INH-HAE in order to validate it for use in this disease.

## Methods

### Study description

#### Psychometric study

**Phase 1** A *post-hoc* analysis of the descriptive characteristics and psychometric properties of the SF-36 was carried out using data provided by an international sample of adult C1-INH-HAE patients who participated in the pilot study for the HAE-QoL development and validation between 2009 and 2011 [5].

**Phase 2** Adult C1-INH-HAE patients in Spain were recruited for the study from the Allergy Department of La Paz University Hospital and the National Association of Patients with Hereditary Angioedema (AEDAF) in order to assess SF-36 reliability (test–retest phase).

### Ethics committee

The study was reviewed and approved by the Clinical Research Ethics Committee of La Paz University Hospital (Madrid, Spain) (PI-281 and PI-1881) and the committees of the participating hospitals according to the specific regulations of each country.

### Participants

Participation in this study was voluntary. Patients were recruited by physicians from our research group in each country. All patients provided informed consent. The inclusion criteria for the patients were that they were at least 18 years old and had a diagnosis of C1-INH-HAE (type I or II) confirmed by a participating physician based on low plasma levels of functional C1 inhibitor and/or low serum antigenic C1 inhibitor. In some cases, mutations in the *SERPING1* gene were also detected to confirm diagnosis [7].

Exclusion criteria were patients under 18 years of age, patients with other types of angioedema, patients having a mental health condition adversely affecting their understanding of the study and/or lack of fluency in the language used to answer the questionnaire.

A convenience sample of patients who were heterogeneous with regard to sex, age, level of education, geographical origin and severity of disease was selected for phase 1, whereas a random sample of patients was obtained for phase 2.

### Questionnaires

The patients filled the following questionnaires in the first phase: a clinical questionnaire on demographic and clinical characteristics (CQ-HAE) [5], the SF-36, and the HAE-QoL v2.0. Validated versions of HAE-QoL and CQ-HAE questionnaires were available in each of the target languages spoken in the participating countries [5]. Validated versions of the SF-36 in every language from all the countries were purchased from QualityMetric for use in the study.

In the second phase, patients were initially required to complete the CQ-HAE, the SF-36 and the HAE-QoL v2.0 questionnaires. Seven to ten days later they answered SF-36 questionnaire again, as well as a short version of the clinical questionnaire to assess the patients’ clinical stability between the two tests (CQ-retest).

The SF-36 questionnaire consists of 36 questions (items) which encompass 8 domains: physical functioning (PF), role physical (RP), bodily pain (BP), general health (GH), vitality (VT), social functioning (SF), role emotional (RE) and mental health (MH). Additionally, the SF-36 includes a transition question asking respondents to assess changes in their general health condition compared with the previous year. Although this item is not used to calculate scales it does provide useful information on perceived changes in the health or functioning since the year before completing the SF-36 [15]. The SF-36 questionnaire was not designed to generate a global health index. However, it allows the calculation of two summary scores, namely, the physical component summary (PCS) and the mental component summary (MCS), by combining the scores of several dimensions. A higher SF-36 score indicates higher HRQoL. No missing data were imputed.

HAE-QoL is the first specific HRQoL instrument for adult patients with C1-INH-HAE [5, 6]. It contains 25 items classified under seven HRQoL domains (treatment difficulties, physical functioning and health, disease-related stigma, emotional role and social functioning, concern about offspring, perceived control over illness, and mental health) [5]: Total HAE-QoL score varies between 25 and 135, where a higher score indicts a better HRQoL. HAE-QoL showed strong psychometric properties with a Cronbach’s coefficient of 0.92 and intraclass correlation coefficient of 0.87 [5].

The C1-INH-HAE severity in the last 6 months was measured by the C1-INH-HAE severity score previously described [5] (see Table 1).

**Table 1:** Ad hoc C1-INH-HAE severity score

Severity score	Criteria
Asymptomatic	No angioedema episodes and no long-term prophylactic treatment
Mild	No life-threatening angioedema episodes and no long-term prophylactic treatment and ≤ 3 episodes/last 6 mos
Moderate	No life-threatening angioedema episodes and ≤ 6 episodes/last 6 mos. with long-term prophylactic treatment (except maintenance treatment with pdC1-INH) or > 4–12 episodes/last 6 mos. without long-term prophylactic treatment
Severe	Life-threatening angioedema episodes and/or 6 episodes/last 6 mos. with long-term prophylactic treatment and/or Maintenance treatment with pdC1-INH and/or > 12 episodes/last 6 mos. without long-term prophylactic treatment

*C1-INH-HAE*, Hereditary angioedema due to C1 inhibitor deficiency; *pdC1-INH*, plasma-derived C1 inhibitor concentrate; *SD*, standard deviation. (Copied from Prior et al. [5], with permission)

### Data collection

Patients completed written questionnaires either in-person at the hospital or at home, which were subsequently sent to La Paz University Hospital (Madrid, Spain) for data processing and analysis.

Anonymous data were entered into an Excel database in accordance with the regulations of the Organic Law on Protection of Personal Data (LOPD 15/1999), the applicable law at the time the study was conducted. The assessment of data entry was verified by two researchers.

Data management and analysis were centralised at La Paz University Hospital (Madrid, Spain).

### Statistical analysis

The analysis involved:

1. Descriptive statistics of the items, including missing values, minimum and maximum scores, ceiling effect, floor effect, mean, standard deviation, median, interquartile range, skewness, kurtosis, corrected homogeneity index (CHI), and internal consistency coefficient or Cronbach’s alpha. A ceiling or floor effect was considered to be present if more than 15% of respondents had the lowest or highest possible scores [16, 17].
2. Psychometric properties analysis of the SF-36 by means of the reliability and validity evidence study:
3. **Internal reliability or internal consistency** was assessed by Cronbach’s alpha coefficient [18], for both summaries and domains of the SF-36. Values between 0.70 and 0.95 were considered optimal [16].
4. **Construct validity** was studied by means of the convergent validity analysis with the HAE-QoL, the study of several predefined clinical hypotheses and the discriminant validity analysis among known groups:
  Convergent validity was assessed by calculating the Pearson correlation coefficient between the scores of the physical (PCS) and mental (MCS) component summaries and the 8 domains of the SF-36 and the total score of the HAE-QoL and its domains. Association was deemed to exist when this coefficient was higher than 0.4.
  It was hypothesized “a priori” that a lower SF-36 score (worse HRQoL) would exist for certain patients; those who had ever undergone intubation or a tracheotomy; and patients who were symptomatic, under long-term prophylactic treatment, had received an inappropriate treatment for angioedema attacks (e.g. antihistamines) or had received psychological/psychiatric care for C1-INH-HAE in the last 6 months. In a post hoc analysis, it was decided to further include the hypotheses that patients who had had laryngeal angioedema attacks, had required emergency intervention and had had a higher number of angioedema episodes in the last 6 months would also have lower HRQoL. Construct validity was considered supported if clinically differentiable patient groups had significantly different SF-36 scores in the expected ways in the two summaries and at least 4 out of the 8 domains. The Kruskal–Wallis test and post hoc comparisons were carried out.
  Discriminant validity among known groups: patients were classified into subgroups according to C1-INH-HAE severity in the last 6 months (Asymptomatic, Mild, Moderate and Severe) (Table 1).^5^ The Asymptomatic and Mild subgroups were combined for statistical analysis. Validity of known groups was considered supported if clinically differentiable patient groups had significantly different SF-36 scores in the expected ways.

The Mann-Whitney *U* test and the Student *t* test were used to compare two independent samples, whereas the Kruskal-Wallis test or one-way ANOVA was used for three or more independent samples. In addition, the *post hoc* analysis was carried out adjusted by the Bonferroni correction.

(c) **Reliability:** The test–retest reliability was measured by means of intraclass correlation coefficient (ICC) in a group of subjects considered stable with regard to their personal situation and clinical condition during retest period. ICC was considered acceptable if ≥ 0.7 [16].
(d) **Minimal clinically important difference (MCID):** Two methods based on the distribution of values were used to estimate MCID:
  MCID-1: The half standard deviation (SD) approach, which has been shown to approximate the threshold of discrimination for a clinically meaningful change or difference in PRO scores for patients with chronic diseases [19].
  MCID-2: The standard error of measurement (SEM), which is widely accepted to represent the MCID of an instrument [20, 21], calculated by multiplying the standard deviation of the instrument by the square root of one minus its reliability coefficient [SD*square root (1-reliability)]. ICC was used as the reliability coefficient.

Contrast hypothesis gave a 95% confidence interval. Data analysis was performed using SPSS v.12.0 and STATA v. 12 statistical software.

## Results

### Patient demographic and clinical characteristics

**Phase 1** International study

Three hundred and thirty-two adult patients with C1-INH-HAE participated in the study. There were sufficiently completed data from 290 patients. The countries participating in the multi-centre study were (from highest to lowest representation in the sample) Spain (n = 42), Germany (n = 42), Hungary (n = 38), Brazil (n = 34), Denmark (n = 27), Poland (n = 22), Canada (n = 21), Romania (n = 19), Austria (n = 18), Argentina (n = 16) and Israel (n = 9). Characteristics of the patients included in the study are shown in Table 2.

**Table 2:** Characteristics of international multicenter study (phase I) and the re-test (phase II) sample groups

Characteristics		N total (N/A)
**Phase I**		
**Age (years) (mean ± SD)**	41.5 ± 14.6	290 (0)
**Gender (male /female) n (%)**	90 (31) /200(69)	290 (0)
**C1-INH-HAE type**		270 (20) *
Type I, n (%)	232 (85.9)	
Type II, n (%)	38 (14.1)	
**Age at onset of symptoms (years) (mean ± SD)**	11.8 (9.6)	274 (16)
**Age at HAE diagnosis (years) (mean ± SD)**	26.4 (14.9)	284 (6)
**Delayed HAE diagnosis (years) (mean ± SD)**	14.4 (13,9)	271 (19)
Diagnosis before the onset of symptoms, n (%)*	11 (4)	273 (17)
**C1-INH-HAE severity †**		273 (17)
Asymptomatic, n (%)	10 (3.7)	
Mild, n (%)	62 (22.7)	
Moderate, n (%)	89 (32.6)	
Severe, n (%)	112 (41.0)	
**Intubation/tracheotomy requirement ever in life, n (%)**	34 (11.8)	287 (3)
**Type of residence**		284 (6)
Rural / semi-urban (< 25.000 inhabitants), n (%) saaxvdfavefvfvnnnn9()25.000	125 (44.0)	
Urban (≥ 25.000 inhabitants), n (%)	159 (56,0)	
**Level of education**		290 (0)
No schooling/primary school, n (%)	72 (24,8)	
High school/further studies, n (%)	218 (75.2)	
**Socioeconomic level**		280 (10)
Low / medium low, n (%)	173 (61.79)	
Medium high / high, n (%)	107 (38.21)	
**Number of emergency visits in the last 6 months**		250 (40)
0, n	149	
1–5, n	70	
6–10, n	16	
≥ 11, n	15	
**Number of AE attacks in the last 6 months,**		2592 (0)
Mean ± SD	9.3 ± 14.3	280 (10)
Median (range)	2 (0–111)	
**Maintenance treatment, n (%)**		282 (8)
Yes n (%)	146 (51.8)	
No n (%)	136 (48.2)	
**Type of maintenance treatment**		277 (13)
Attenuated androgens, yes n (%)	101 (36.5)	
Antifibrinolytics, yes n (%)	18 (6.5)	
pdC1INH, yes n (%)	15 (5.4)	
Others (desogestrel), yes n (%)	2 (0.7)	
Several, yes n (%)	5 (1.7)	
**Inadequate medical treatment of AE episodes in the last 6 months n (%)**	34 (12.3)	276 (14)
**Psychiatric/psychological care or** **treatment (in the last 6 months), n (%)**	31 (11.1)	279 (11)
**pdC1INH at home**		282 (8)
No + not available n (%)	96 (34)	
Yes + not self-administered n (%)	143 (50.7)	
Yes + yes self-administered n (%)	43 (15.2)	
**Phase II**		
**Age (years) (mean ± SD)**	52.4 ± 13.0	20 (0)
**Gender (male /female) n (%)**	10 (50) /10(50)	20 (0)
**C1-INH-HAE type**		20 (0)
Type I, n (%)	14 (70)	
Type II, n (%)	3 (15)	
Don´t know	3 (15)	
**Age at onset of symptoms (years) (mean ± SD)**	20.1 (11.7)	20 (0)
**Age at HAE diagnosis (years) (mean ± SD)**	30.2 (18.7)	20 (0)
**Family history of C1-INH-HAE, n (%)**	16 (80)	20 (0)
**Family death from suffocation, n (%)**	5 (25)	20 (0)
**Intubation/tracheotomy requirement, n (%)**	1 (5)	20 (0)

*AE*: angioedema; *C1-INH-HAE*: hereditary angioedema due to C1-inhibitor deficiency; *pdC1-INH*: plasma-derived C1 inhibitor concentrate

^*^Physicians selected patients with laboratory-confirmed diagnosis of AEH-C1-INH. However, some respondents of the self-administered clinical questionnaire were unable to identify which type of C1-INH-HAE they had. † According to the severity score created ad hoc [5];

*SD*: standard deviation

### Psychometric analysis of SF-36

The descriptive study of the SF-36 items can be seen in Table 3. The CHI of individual items varied between 0.489 and 0.880. The non-response rate per item was very low and varied from 1 to 3.4%. Two hundred and sixty-six patients (91.7%) had no missing data. The item ceiling effect was present in 25 out of the 35 items included in the SF-36. This ceiling effect was very high, mainly in the domains “PF” (items SF3b to SF3j, which varied between 65.0% and 89.5%), “RE” (items SF5a to SF5c, ranging between 48.6% and 52.6%) and “RP” (items SF4a to SF4d, with a fluctuation between 38.2% and 46.5%). The SF9c item of the “MH” domain also showed a significant ceiling effect (50.2%). The only domain in which no item had a ceiling effect was “VT” with 4 items (SF9a, SF9e, SF9g, SF9i). In general, the floor effect was very low, with only 3/35 items with minor floor effect (between 17.3 and 27.9%). The only items with a floor effect were SF3a in the “PF” domain (27.9%), and SF11b and SF11d in the “GH” domain with 17.3% and 21.2%, respectively.

**Table 3:** Descriptive analysis of the SF-36 item scores

General group of items n = 35	Item no	No answers (%)	Min–max Value	Floor effect (%)	Ceiling effect (%)	Mean	Standard deviation	Corrected homogeneity index	Cronbach’s α
Physical functioning	SF3a	1.0	1–3	27.9	36.2	2.08	0.80	0.57	0.90
Physical functioning	SF3b	1.4	1–3	6.3	65.0	2.59	0.61	0.71	
Physical functioning	SF3c	1.4	1–3	7.0	66.8	2.60	0.62	0.63	
Physical functioning	SF3d	3.4	1–3	7.9	66.4	2.59	0.63	0.74	
Physical functioning	SF3e	2.8	1–3	1.4	88.3	2.87	0.38	0.67	
Physical functioning	SF3f	1.7	1–3	6.3	72.6	2.66	0.59	0.64	
Physical functioning	SF3g	1.4	1–3	7.3	73.1	2.66	0.61	0.77	
Physical functioning	SF3h	1.7	1–3	8.1	78.2	2.70	0.61	0.71	
Physical functioning	SF3i	1.7	1–3	3.5	88.4	2.85	0.45	0.63	
Physical functioning	SF3j	1.4	1–3	2.4	89.5	2.87	0.40	0.51	
Role physical	SF4a	1.7	1–5	3.2	43.9	3.95	1.13	0.82	0.93
Role physical	SF4b	1.7	1–5	3.2	38.2	3.80	116	0.89	
Role physical	SF4c	1.7	1–5	3.5	45.6	3.93	1.19	0.84	
Role physical	SF4d	1.4	1–5	3. 8	46.5	3.95	1.17	0.83	
Bodily pain	SF7*	1.0	1–6	4.9	25.8	4.9	1.56	0.87	0.91
Bodily pain	SF8*	1.7	1–6	6.0	36.5	3.92	1.59	0.87	
General health	SF1*	1.7	1–5	6.0	6.7	3.07	1.07	0.65	0.82
General health	SF11a	2.1	1–5	14.4	29.2	3.24	1.46	0.57	
General health	SF11b*	2.4	1–5	17.3	13.4	2.94	1.32	0.65	
General health	SF11c	2.1	1–5	3.5	30.6	3.57	1.16	0.49	
General health	SF11d*	2.4	1–5	21.2	9.5	2.85	1.34	0.72	
Vitality	SF9a*	1.7	1–5	4.2	11.2	3.44	1.01	0.62	0.82
Vitality	SF9e*	1.4	1–5	7.3	6.6	3.17	1.05	0.64	
Vitality	SF9g	1.4	1–5	4.2	14.3	3.33	1.04	0.65	
Vitality	SF9i	1.4	1–5	9.1	4.5	2.96	0.98	0.63	
Social functioning	SF6*	1.4	1–5	2.4	45.8	3.97	1.16	0.70	0.82
Social functioning	SF10*	1.7	1–5	2.1	36.1	3.93	1.01	0.70	
Role emotional	SF5a	1.7	1–5	2.5	52.6	4.13	1.09	0.86	0.92
Role emotional	SF5b	2.1	1–5	3.2	48.6	4.07	1.10	0.88	
Role emotional	SF5c	1.7	1–5	2.5	49.8	4.11	1.06	0.76	
Mental health	SF9b	1.0	1–5	5.6	17.8	3.45	1.11	0.69	0.87
Mental health	SF9c	1.7	1–5	1.8	50.2	4.18	1.01	0.74	
Mental health	SF9d*	2.4	1–5	4.9	5.7	3.20	1.01	0.69	
Mental health	SF9f	1.4	1–5	1.7	29.4	3.82	0.99	0.70	
Mental health	SF9h*	1.7	1–5	3.5	13.7	3.52	0.99	0.62	

^*^ Items *SF1, SF6, SF7, SF8, SF9a, SF9d, SF9g, SF9h, SF10, SF11b, SF11d* were recoded as indicated in the Methods section

Regarding the SF-36v2 domains, no floor effect was observed in the domains, while a moderate ceiling effect was observed in 5 out of 8 domains: 31.8% for PF; 30.8% for “RP”; 24.8% for “BP”; 32.9% for “SF” and 41.0% for “RE” (see Table 4).

**Table 4:** Descriptive analysis of the SF-36 dimension scores

Domains	Nr items	Min value	Max value	Floor effect (%)	Ceiling effect (%)	Mean	SD	p25	p50 (median)	p75	Skewness	Kurtosis	Cronbach’s α
Physical functioning	10	10	30	0.7	31.8	26.53	4.15	24	28	30	− 1.457	1.931	0.90
Role physical	4	4	20	2.0	30.8	15.66	4.22	12	16	20	− .714	− .333	0.93
Bodily pain	2	2	12	3.1	24.8	8.01	3.03	5.88	8.2	10.8	− .104	− 1.129	0.93
General health	5	5	25	0.7	2.1	15.79	4.86	12	16	19.4	− .061	− .829	0.82
Vitality	4	4	20	0.0	1.7	12.94	3.25	11	13	15	− .254	− 496	0.82
Social functioning	2	2	10	1.0	32.9	7.91	2.00	6	8	10	− .640	− .519	0.82
Role emotional	3	3	15	1.4	41.0	12.32	3.01	10	13	15	− .966	.147	0.92
Mental health	5	5	25	0.0	3.1	18.23	4.18	16	19	21	− .597	− .363	0.87

SD: standard deviation

The SF-36 showed an internal consistency from good to excellent. Cronbach’s alpha coefficient varied from 0.82 to 0.93 for the domains (Table 4).

In the convergent validity study, the summaries of the physical component (PCS) and mental component (MCS) of the SF-36 showed a good correlation with the HAE-QoL total score (0.45 and 0.64 respectively, *P* < 0.001). The MCS presented higher correlations with all the HAE-QoL domains than the PCS. The lowest correlation was found between the “Concern about offspring” domain of the HAE-QoL with both summaries of the SF-36 (MCS 0.30 and PCS 0.17).

Similarly, the total score of the HAE-QoL showed a good correlation with the “PF” (0.64), “SF” (0.59) and “GH” (0.58) domains of the SF-36. Furthermore, statistically significant mild-to-moderate correlations (≥ 0.4) were observed among most of the SF-36 and the HAE-QoL domains, except for the “Concern about offspring” domain of the HAE-QoL, with which all the correlations were < 0.4. Moreover, a mild correlation was observed (< 0.4) between the “PF” domain of the SF-36 and the “Disease-related stigma” (0.35), “Treatment difficulties” (0.38) and “Perceived control over disease” (0.39) domains of the HAE-QoL and between “VT” of the SF-36 and “Disease-related stigma” of the HAE-QoL (0.39). These results have been previously reported [5] and are shown in (see Table 5).

**Table 5:** Convergent validity of the SF-36 with the HAE-QoL

Subscales HAE-QoL	SF-36									
	PF	RP	BP	GH	V	SF	RE	MH	PCS	MCS
Treatment difficulties	0.38	0.50	0.55	0.42	0.43	0.50	0.44	0.48	0.38	0.50
Physical functioning and health	0.40	0.60	0.60	0.52	0.46	0.52	0.43	0.50	0.40	0.60
Disease-related stigma	0.35	0.51	0.51	0.47	0.39	0.49	0.40	0.43	0.35	0.51
Emotional role and social functioning	0.42	0.59	0.56	0.51	0.50	0.59	0.45	0.50	0.42	0.59
Concern about offspring	0.17	0.30	0.36	0.33	0.28	0.30	0.27	0.30	0.17	0.30
Perceived control over illness	0.39	0.55	0.54	0.50	0.44	0.44	0.47	0.46	0.39	0.55
Mental health	0.42	0.57	0.58	0.54	0.51	0.54	0.49	0.58	0.42	0.57
Total HAE-QoL	0.42	0.64	0.64	0.58	0.53	0.59	0.52	0.57	0.45	0.64

*SF-36*, Short Form 36-item Health Survey Version 2.0; *HAE-QoL*, Hereditary angioedema quality of life; *PF*, physical functioning; *RP*, role functioning; *BP*, bodily pain; *GH*, general health perceptions; *V*, vitality; *SF*, social functioning; *RE*, role emotional; *MH*, mental health; *PCS*, physical component summary;

*MCS*, component mental summary; *Pearson correlation coefficient*: All correlations were statistically significant with *P* < .001, except for

correlation between “*Perceived control over illness*” from *HAE-QoL* and “*Physical function*” from *SF-36* that was *P* = 0.004

Construct validity based on combined a priori predefined and post hoc defined hypotheses according to clinical criteria was confirmed in 7 out of the 8 hypotheses. Four out of the eight hypotheses (50%) showed significant differences in all the domains and the two summaries, three in the two summaries and at least 4 of the domains, and only one of the hypotheses was not satisfied. Details are summed up in Table 6.

**Table 6:** Construct validity according to predefined hypotheses regarding clinical criteria

		PF	RP	BP	GH	V	SF	RE	MH	PSC	MSC
ASYMPTOMATIC ^*^ (missing n = 17)	NO (n = 10) Mean ± DS	82.3 ± 21,0	72.1 ± 26.6	58.9 ± 30.3	53 ± 24.2	55.1 ± 20.3	73.2 ± 25.1	77.4 ± 25.1	65.3 ± 20.6	49.6 ± 8.8	45.9 ± 10.4
	YES (n = 263) Mean ± SD	90.6 ± 16.1	95.3 ± 7.3	85.8 ± 22.8	72.1 ± 22.2	72.7 ± 19.2	87.5 ± 20.0	89.6 ± 19.8	84.4 ± 12.7	53.1 ± 6.8	55.0 ± 2.9
	P	0.12	**0.01**	**0.02**	**0.04**	**0.02**	0.09	0.11	**0.00**	0.12	**0.01**
INTUBATION / TRACHEOTOMY SOMETIMES IN LIFE ^*^ (missing n = 3)	NO (n = 253) Mean ± SD	83.5 ± 19.6	73.6 ± 25.5	60.6 ± 30.0	54.6 ± 24.0	55.9 ± 20.4	74.6 ± 24.0	78.8 ± 23.6	66.2 ± 20.5	50.1 ± 8.2	46.5 ± 10.0
	YES (n = 34) Mean ± SD	74.7 ± 29.5	66.2 ± 31.9	52.6 ± 32.6	45.1 ± 24.8	54.0 ± 21.2	68.8 ± 28.9	70.1 ± 32.2	64.5 ± 21.1	46.4 ± 12.4	43.6 ± 12.5
	P	0.12	0.13	0.16	**0.02**	0.62	0.28	0.15	0.67	0.12	0.13
LONG TERM PROFILAXIS ^†^ (missing n = 8)	NO (n = 136) Mean ± SD	85.3 ± 20.0	75.1 ± 26.7	66.5 ± 29,2	58.3 ± 22.6	57.9 ± 20.0	76.1 ± 25.4	79.5 ± 25.7	69.7 ± 20.6	50.9 ± 8.4	47.1 ± 10.5
	YES (n = 146) Mean ± SD	79.6 ± 21.6	70.2 ± 26.3	53.9 ± 30.2	48.9 ± 25.3	53.7 ± 20.7	71.4 ± 24.5	75.6 ± 24.6	62.2 ± 19.8	48.4 ± 9.1	45.2 ± 10.3
	P	**0.02**	0.13	**0.00**	**0.00**	**0.08**	0.12	0.21	**0.00**	**0.02**	0.13
INAPPROPRIATE MEDICAL TREATMENT ^†‡^ (missing n = 14)	NO (n = 242) Mean ± SD	84.4 ± 20.0	75.1 ± 25.2	62.7 ± 30.1	55.6 ± 23.8	57.0 ± 20.6	76.3 ± 23.8	79.3 ± 24.5	67.7 ± 19.6	50.5 ± 8.4	47.1 ± 9.9
	YES (n = 34) Mean ± SD	69.8 ± 23.0	56.1 ± 29.9	41.8 ± 26.2	39.7 ± 23.0	45.6 ± 18.1	56.3 ± 27.4	65.4 ± 27.3	55.6 ± 23.9	44.3 ± 9.7	39,6 ± 11,7
	P	**0.00**	**0.00**	**0.00**	**0.00**	**0.00**	**0.00**	**0.00**	**0.01**	**0.00**	**0.00**
PSYCHIATRIC/PSYCHOLOGICAL CARE OR TREATMENT) ^†^ (missing n = 11)	NO (n = 263) Mean ± SD	83.8 ± 20.6	75.6 ± 25.6	62.9 ± 30.1	56.1 ± 23.4	57.1 ± 20.1	76.6 ± 23.9	80.8 ± 23.6	68.5 ± 19.4	50.2 ± 8.8	47.3 ± 10.0
	SI (n = 18) Mean ± SD	73.2 ± 22.6	50.7 ± 24.5	36.0 ± 20.0	34.0 ± 22.5	43.8 ± 17.1	52.1 ± 24.1	54.6 ± 26.2	46.6 ± 19.3	45.8 ± 9.5	37.5 ± 9.6
	P	**0.01**	**0.00**	**0.00**	**0.00**	**0.00**	**0.00**	**0.00**	**0.00**	**0.01**	**0.00**
LARYNX ATTACKS IN THE LAST 6 MONTHS ^†^ (missing n = 9)	No (n = 230)	84.6 ± 20.0	74.7 ± 25.9	62.0 ± 30.1	54.7 ± 23.8	56.4 ± 20.5	75.4 ± 23.8	79.1 ± 24.2	67.3 ± 19.7	50.6 ± 8.4	46.9 ± 10.2
	Yes (n = 51)	71.1 ± 22.3	61.4 ± 27.4	49.3 ± 29.2	46.4 ± 25.8	50.6 ± 20.4	65.3 ± 28.9	70.4 ± 28.3	58.9 ± 23.9	44.9 ± 9.4	41.7 ± 10.7
	P	** < 0.001**	**.001**	**.008**	**.029**	.072	**.024**	**.028**	**.025**	** < 0.001**	**0.001**
EMERGENCY VISITS IN THE LAST 6 MONTHS (missing n = 40)	0 (n = 149)	84.6 ± 19.7	80.0 ± 24.3	66.5 ± 27.8	58.5 ± 24.1	58.8 ± 20.5	79.2 ± 24.0	80.4 ± 24.7	67.9 ± 20.3	50.5 ± 8.3	49.0 ± 9.5
	1–5 (n = 70)	82.8 ± 20.8	67.7 ± 26.1	54.2 ± 28.5	49.1 ± 23.2	55.0 ± 20.7	70.1 ± 24.6	79.1 ± 22.9	66.1 ± 19.6	49.8 ± 8.8	44.2 ± 10.2
	6–10 (n = 16)	73.3 ± 21.0	50.0 ± 16.2	26.4 ± 19.4	44.1 ± 26.2	40.0 ± 19.5	51.6 ± 12.8	66.1 ± 23.7	56.0 ± 25.4	45.8 ± 8.8	37.3 ± 6.4
	≥ 11 (n = 15)	73.3 ± 24.4	57.1 ± 27.5	38.9 ± 34.8	40.1 ± 23.9	45.0 ± 17.6	60.8 ± 30.6	65.0 ± 29.2	49.3 ± 21.1	45.81 ± 10.27	40.0 ± 10.8
	P	**.009**	**.000**	**.000**	**.002**	**.001**	**.000**	**.018**	**.006**	**.009**	**.000**
N ATTACKS IN THE LAST 6 M (missing 10)	0	86.0 ± 21.1	84.6 ± 22.0	74.0 ± 28.7	65.6 ± 23.4	61.2 ± 22.9	81.8 ± 23.0	82.2 ± 24.5	71.4 ± 20.6	51.1 ± 8.9	50.8 ± 8.6
	1–5	83.9 ± 19.9	77.7 ± 26.7	68.5 ± 27.7	57.6 ± 23.1	60.0 ± 18.4	79.7 ± 22.4	81.3 ± 25.3	70.2 ± 18.0	50.3 ± 8.4	48.1 ± 10.5
	6–10	78.5 ± 22.4	64.4 ± 21.7	53.4 ± 28.3	40.8 ± 21.6	47.6 ± 18.8	65.5 ± 25.6	73.7 ± 23.6	58.9 ± 20.7	48.0 ± 9.4	42.9 ± 8.5
	≥ 11	78.9 ± 21.7	60.3 ± 25.9	40.8 ± 26.1	44.6 ± 22.6	48.8 ± 19.9	62.7 ± 25.1	70.9 ± 25.0	59.1 ± 22.1	48.13 ± 9.13	41.3 ± 10.1
	P	**.020**	**.000**	**.000**	**.000**	**.000**	**.000**	**.005**	**.000**		**.000**

^*^ *Mann–Whitney test*; † *Student t test*, ‡ *antiallergic* treatment for attacks in the last 6 months; PCS: physical component summary; *MCS*, mental component summary; *PF*, physical functioning; *SF*, social functioning; *RP*, role limitations due to physical health problems; *RE*, role limitations due to emotional problems; *MH*, mental health; *V*, vitality; *BP*, bodily pain; *GH*, general health perceptions; *SD*, Standard deviation

In the discriminant validity assessment, significant differences were observed in both SF-36 summaries and all SF-36 domain scores among the 3 categories of the C1-INH-HAE severity scale (Asymptomatic-Mild, Moderate, Severe) (see Table 7).

**Table 7:** Discriminant validity between known groups of the SF-36

Dimensions of the SF-36	Description	C1-INH-HAE severity			*P*	*P* values for two to two post hoc comparisons (Bonferroni Correction)		
		Asymptomatic / mild	Moderate	Severe		A/M–S	A/M- Mod	Mod-S
Physical functioning	Mean ± SD	89.5 ± 17.3	81.4 ± 19.8	77.6 ± 23.4	** < 0.001**	** < 0.001**	1.000	**.012**
	Median (range)	95 (86.2–1000)	90 (65–100)	85 (65–95)				
	n	72	87	106				
Role Physical	Mean ± SD	82.8 ± 25.9	74.6 ± 23.3	62.9 ± 26.8	** < 0.001**	** < 0.001**	.760	.053
	Median (range)	100 (75–100)	75 (56.3–100)	63,5 (43.8–87.5)				
	n	73	89	108				
Bodily pain	Mean ± SD	77.0 ± 25.2	60.7 ± 29.6	46.1 ± 27.9	** < 0.001**	** < 0.001**	.876	**.006**
	Median (range)	84 (61–100)	62 (41–84)	41 (22–62)				
	n	73	88	109				
General health	Mean ± SD	64.3 ± 20.8	54.4 ± 25.0	44.3 ± 23.2	** < 0.001**	** < 0.001**	1.000	.077
	median (range)	67 (52–80)	56 (30.5–72)	42 (30–62)				
	n	73	88	107				
Vitality	Mean ± SD	63.2 ± 20.1	55.5 ± 18.4	49,8 ± 20,7	** < 0.001**	** < 0.001**	.379	.059
	Median (range)	62.5 (53.1–75)	56.3 (43.8–68.8)	50 (31.3–68.8)				
	n	73	88	110				
Social functioning	Mean ± SD	80.6 ± 23.4	77,1 ± 22,7	65,6 ± 25,9	** < 0.001**	** < 0.001**	.850	1.000
	Median (range)	87.5 (75–100)	87,5 (62,5–100)	62,5 (50–87,5)				
	n	72	88	109				
Role Emotional	Mean ± SD	80.9 ± 24.7	81.9 ± 23.4	71.0 ± 26.1	**.005**	**.020**	.606	1.000
	Median (range)	91.7 (66.7–100)	91.7 (75–100)	75 (50–100)				
	n	73	89	108				
General health	Mean ± SD	71.8 ± 20.4	67.4 ± 17.7	60.0 ± 21.5	** < 0.001**	** < 0.001**	**.048**	.600
	Median (range)	75 (65–85)	67.5 (56.3–83,8)	65 (45–75)				
	n	71	88	108				
PCS	Mean ± SD	52.6 ± 7.3	49.2 ± 8.3	47.6–9.8	** < 0.001**	** < 0.001**	1.000	**.011**
	median (range)	54.9 (51.2–57.0	52.8 (42.3–57.0)	50.7 (42.3–54.9)				
	n	72	87	106				
MCS	Mean ± SD	50.1 ± 10.2	46.9 ± 9.1	42.3 ± 10.5	** < 0.001**	** < 0.001**	.760	.052
	Median (range)	56.9 (47.1–56.9)	47.1 (39.7–56.9)	42.2 (34.8–52.0)				
	n	73	89	108				

*SD*, standard deviation; *P,* statistically significant *P* values are highlighted in red; A/M, Asymptomatic/mild; Mod, moderate; S, severe

**Phase 2** Thirty-seven adult patients with C1-INH-HAE participated in the test–retest reliability study (phase 2 of the psychometric study). Thirty patients had all data completed and 20 of them were considered stable. The demographic and clinical characteristics of these patients are shown in Table 2.

The ICC (95% confidence interval) can be seen in Table 8 and varied between 0.758 for the “VT” domain and 0.962 for the “SF” domain.

**Table 8:** Intraclass correlation coefficient (phase 2: test–retest)

Physical functioning	ICC	Confidence level at 95%	
	0.949	0.862	0.981
Role physical	0.912	0.788	0.965
Bodily pain	0.811	0.576	0.922
General health	0.903	0.759	0.962
Vitality	0.758	0.469	0.900
Social functioning	0.962	0.906	0.985
Role emotional	0.913	0.791	0.965
Mental health	0.876	0.706	0.950
Physical component summary physicalPhysical component ssummary	0.922	0.809	0.969
Mental component summary component	0.915	0.793	0.966

*ICC*, Intraclass Correlation Coefficient

The MCID for the different domains and the two component summaries of the SF-36 is shown in Table 9.

**Table 9:** Estimation of the minimal clinical important difference

Domain/Summary	SD	ICC	MCID-1 (SD/2)	MCID-2 (SEM)
PF	4.18	0.949	2.09	0.94
RP	4.24	0.912	2.12	1.26
BP	3.04	0.811	1.52	1.32
GH	4.86	0.903	2.43	1.51
VT	3.27	0.758	1.64	1.61
SF	2.00	0.962	1.00	0.39
RE	3.00	0.913	1.50	0.88
MH	4.13	0.876	2.06	1.45
PCS	8.80	0.922	4.40	2.46
MCS	10.38	0.915	5.19	3.03

*MCID-1* was calculated as half of the standard deviation*. MCID-2* was assumed to be the *SEM,* which was calculated multiplying the standard deviation of the instrument by the square root of one minus its reliability coefficient [SD*square root (1-reliability)]. Intraclass correlation coefficient (ICC) was used as the reliability coefficient*. BP:* Bodily pain*; GH:* General health*; ICC*: intraclass correlation coefficient*; MCID:* minimal clinically important difference*; MCS:* Mental component summary*; MH:* Mental health; *PCS:* Physical component summary*; PF:* Physical functioning*; RE:* Role emotional*; RP:* Role physical*; SD:* standard deviation*; SEM:* standard error of measurement*; SF: Social functioning; VT: Vitality*

## Discussion

The SF-36 is one of the most commonly used generic HRQoL questionnaires worldwide, in studies that measure the impact of a disease on HRQoL in different groups of patients [22–31], as well as studies that assess the effect of certain therapeutic interventions on HRQoL [32–38]. It has also been used as a reference in the validation of new instruments [39–44]. The SF-36 was used to measure HRQoL in patients with C1-INH-HAE [25–31] and to assess the effect of some therapeutic interventions [35, 36, 38, 45–47]. However, we have found no evidence of any studies on its psychometric properties in patients with C1-INH-HAE and, to the best of our knowledge, it has yet to be for use in C1-INH-HAE.

The psychometric analysis in this study yields satisfactory results overall and provides support for validating the SF-36 as a tool for assessing HRQoL in C1-INH-HAE patients. The SF-36 showed good internal consistency, with all Cronbach’s α coefficient values being higher than 0.7. Similar data were observed for the eight domains in other studies [42–44, 48].

The extremely low rate of unanswered questions indicates the questionnaire was suitable for patients with C1-INH-HAE. However, further analysis reveals elevated ceiling effect in the majority of individual items. This suggests that either a greater choice of answers should be included at the top of the scale or respondents did not consider those items to be relevant to C1-INH-HAE. In either case, it would clearly limit the content validity of the SF-36 in C1-INH-HAE.

The ceiling effect is present in 5 out of the 8 SF-36 domains (“RE”, “SF”, “PF”, “RP”, “BP”). However, we should take into account that we adopted a very strict definition of this effect (if > 15% of respondents obtained the highest possible score), in comparison to other studies in which the threshold was as high as 60% [49]. The presence of the ceiling effect indicates that there may be a lack of response options for items at the top of the scale, which would imply a limited content validity. Consequently, patients with the highest score may not be distinguished apparently and thus reliability would be reduced. It could also indicate that these domains are not relevant to C1-INH-HAE patients. On the contrary, no floor effect was found in the SF-36 domains, which might mean there is not a lack of responses at the bottom of the scale. The SF-36 has certain content validity limitations that may affect its use in C1-INH-HAE. Similar findings have already been described in a study in which the author found a low sensitivity of the SF-36 when assessing subtle variations of functional status and emotional functioning in patients with brain tumors [50].

As there is no single gold standard assessment tool for HRQoL, we analysed convergent criterion validity by comparing data from the SF-36 and HAE-QoL questionnaires. In our study, correlations obtained among the SF-36 domains and summary scores, and the HAE-QoL total and domain scores were mostly mild to moderate (> 0.40) and statistically significant, which indicates some agreement between the two instruments. The strongest correlations were seen between the HAE-QoL total score and the “BP” and “RP” domains, as well as the “MCS” of the SF-36. Higher correlations were also observed among related domains of both questionnaires (such as the “MH” domain of both questionnaires, “Physical functioning and health” with “RP” and “Emotional and Social roles” with “SF”) than among other unrelated domains. Based on these results, we can assume that coherence and equivalence are verified for the quality-of-life concept, as assessed by these two instruments. This indicates that both scales coincide in subjective and objective aspects that make up the construct, although their conceptual structures and items differ. Furthermore, the lack of strong correlations might be due to the fact that SF-36 is a generic questionnaire while the HAE-QoL is specifically for patients with C1-INH-HAE. This would also explain the low correlations observed between the “Concern about offspring” domain and the SF-36 domains and their physical and mental summaries, as this aspect is specific for C1-INH-HAE and other hereditary diseases and could not be adequately considered by a generic questionnaire such as the SF-36.

For the construct validity, the recommended quality criterion that at least 75% of pre-established hypotheses be confirmed [16] was fulfilled using the combination of “a priori” and “post hoc” defined criteria with an 87.5% (7/8) of confirmed hypothesis. It is worth noting that patients who presented some factors which could be a priori considered determinants of the impact on HRQoL (such as having undergone intubation or a tracheotomy at least once) showed no significant differences. Past intubation or tracheotomy procedures may have no impact on current HRQoL as they may have been performed years earlier and, as a result, are no longer of concern at the time of questioning. Therefore, it would not be a good criterion on which to assess the construct validity of the instrument. This issue also arose in the psychometric study of the HAE-QoL [5]. With respect to other factors, such as the effect of long-term prophylaxis (LTP), no significant differences were observed in the “RP”, “RE”, and “SF” domains, in which there was a ceiling effect, and in the “VT” domain, which had neither floor nor ceiling effects. The variable of having angioedema symptoms in the last 6 months had no significant differences in the “PF”, “SF”, and “RE” domains, and all of them exhibited a ceiling effect.

Analysis of the discriminant validity of the SF-36, shows discrimination was good among patients with different levels of C1-INH-HAE severity in the last 6 months. There were significant differences in the 3 scoring groups across all domains and the two summaries, with HRQoL lower when the severity of the disease was higher. Such data show the SF-36 capacity to distinguish among these known groups.

An examination of test–retest reliability shows that the generic SF-36 questionnaire is stable in patients with C1-INH-HAE, as it meets the recommended standards of the GA2LEN taskforce for assessing Patient-Reported Outcomes on allergy [51]. This means that SF-36 is stable in patients with C1-INH-HAE.

The MCID calculated by two different distribution methods shows that the generic SF-36 questionnaire could be useful as a tool for detecting real changes in HRQoL in patients with C1-INH-HAE. MCID has been evaluated to a lesser degree than other psychometric properties in other studies that validate SF-36 in other diseases.

The main limitations of the study include the post hoc design of the study and the different sample sizes among participating countries.

Despite these disadvantages, the internationally accepted scientific recommendations for the validation of HRQoL measurement instruments have been followed [15, 17], and data on reliability and content and construct validity have been highly acceptable. Moreover, as an international multicentric study, it provides results on which to base generalization, unlike studies with less diverse patient samples.

## Conclusions

This is the first study to assess the psychometric properties of a generic instrument, the SF-36, in adult patients with C1-INH-HAE.

The SF-36 psychometric properties have shown that it has a limited content validity, revealing with a high ceiling in many of the items and in several domains. Despite this limitation, it shows no floor effect and has a high internal consistency, together with good construct validity and high reliability and reproducibility in C1-INH-HAE.

This validation will facilitate the interpretation of HRQoL studies performed using the SF-36 in adult C1-INH-HAE patients and lays the groundwork for future studies on how C1-INH-HAE affects HRQoL in comparison with other diseases in which SF-36 is used to assess HRQoL.

## Data Availability

The datasets collected and/or analyzed during the current study are available from the corresponding author upon reasonable request.

## Abbreviations

AEDAF: Spanish Association of Familial Angioedema (Asociación Española de Angioedema Familiar)
BP: Bodily pain
CHI: Corrected homogeneity index
CQ-HAE: Clinical questionnaire on hereditary angioedema
CQ-retest: Clinical questionnaire for retest phase
C1-INH-HAE: Hereditary angioedema due to C1-inhibitor deficiency
GH: General health
HRQoL: Health-related quality of life
HAE-QoL: Hereditary Angioedema Quality of Life
ICC: Intraclass correlation coefficient
LTP: Long term prophylaxis
MCID: Minimal clinically important difference
MCS: Mental component summary
MH: Mental health
PCS: Physical component summary
PF: Physical functioning
RE: Role emotional
RP: Role physical
SD: Standard deviation
SEM: Standard error of measurement
SF: Social functioning
SF-36v2: The 36-item Short-Form Health Survey
VT: Vitality

## References

[1] Farkas H, Martinez-Saguer I, Bork K, Bowen T, Craig T, Frank M (2017). International consensus on the diagnosis and management of pediatric patients with hereditary angioedema with C1 inhibitor deficiency. Allergy.

[2] Cicardi M, Zuraw BL (2018). Angioedema Due to Bradykinin Dysregulation. J Allergy Clin Immunol Pract.

[3] Aygören-Pürsün E, Magerl M, Maetzel A, Maurer M (2018). Epidemiology of Bradykinin-mediated angioedema: a systematic investigation of epidemiological studies. Orphanet J Rare Dis.

[4] Roche O, Blanch A, Caballero T, Sastre N, Callejo D, López-Trascasa M (2005). Hereditary angioedema due to C1 inhibitor deficiency: patient registry and approach to the prevalence in Spain. Ann Allergy Asthma Immunol.

[5] Prior N, Remor E, Pérez-Fernández E, Caminoa M, Gómez-Traseira C, Gayá F (2016). Psychometric Field Study of Hereditary Angioedema Quality of Life Questionnaire for Adults: HAE-QoL. J Allergy Clin Immunol Pract.

[6] Prior N, Remor E, Gómez-Traseira C, López-Serrano C, Cabañas R, Contreras J (2012). Development of a disease-specific quality of life questionnaire for adult patients with hereditary angioedema due to C1 inhibitor deficiency (HAE-QoL): Spanish multi-centre research project. Health Qual Life Outcomes.

[7] Longhurst H, Cicardi M (2012). Hereditary angio-oedema. Lancet.

[8] Bygum A, Aygören-Pürsün E, Caballero T, Beusterien K, Gholizadeh S, Musingarimi P (2012). The hereditary angioedema burden of illness study in Europe (HAE-BOIS-Europe): background and methodology. BMC Dermatol.

[9] Bygum A, Aygören-Pürsün E, Beusterien K, Hautamaki E, Sisic Z, Wait S (2015). Burden of illness in hereditary angioedema: a conceptual model. Acta Derm Venereol.

[10] Caballero T, Aygören-Pürsün E, Bygum A, Beusterien K, Hautamaki E, Sisic Z (2014). The humanistic burden of hereditary angioedema: results from the Burden of Illness Study in Europe. Allergy asthma Proc.

[11] Caballero T, Prior N (2017). Burden of illness and quality-of-life measures in angioedema conditions. Immunol Allergy Clin N Am.

[12] Maurer M, Magerl M, Ansotegui I, Aygören-Pürsün E, Betschel S, Bork K (2018). The international WAO/EAACI guideline for the management of hereditary angioedema-The 2017 revision and update. Allergy.

[13] Ware JE, Sherbourne CD (1992). The MOS 36-item short-form health survey (SF-36). I. Conceptual framework and item selection. Med Care.

[14] Garratt A, Schmidt L, Mackintosh A, Fitzpatrick R (2002). Quality of life measurement: bibliographic study of patient assessed health outcome measures. BMJ.

[15] Vilagut G, Ferrer M, Rajmil L (2005). El cuestionario de salud SF-36 español: una década de experiencia y nuevos desarrollos. Gac Sanit.

[16] Terwee CB, Bot SDM, de Boer MR, van der Windt DAWM, Knol DL, Dekker J (2007). Quality criteria were proposed for measurement properties of health status questionnaires. J Clin Epidemiol.

[17] Mchorney CA, Ware JE, Raczek AE (1993). The MOS 36-Item Short-Form Health Survey (SF-36): II. Psychometric and Clinical Tests of Validity in Measuring Physical and Mental Health Constructs.

[18] Cronbach LJ (1951). Coefficient alpha and the internal structure of tests. Psychometrika.

[19] Norman GR, Sloan JA, Wyrwich KW (2003). Interpretation of changes in health-related quality of life. Med Care.

[20] Wyrwich KW, Nienaber NA, Tierney WM, Wolinsky FD (1999). Linking clinical relevance and statistical significance in evaluating intra-individual changes in health-related quality of life. Med Care.

[21] Wyrwich KW, Tierney WM, Wolinsky FD (1999). Further Evidence Supporting an SEM-based criterion for identifying meaningful intra-individual changes in health-related quality of life. J Clin Epidemiol.

[22] Badia X, Mearin F, Balboa A, Baró E, Caldwell E, Cucala M (2002). Burden of illness in irritable bowel syndrome comparing Rome I and Rome II criteria. Pharmacoeconomics.

[23] Rebollo P, Ortega F, Baltar JM, Alvarez-Ude F, Alvarez Navascués R, Alvarez-Grande J (2001). Is the loss of health-related quality of life during renal replacement therapy lower in elderly patients than in younger patients?. Nephrol Dial Transplant.

[24] Domingo-Salvany A, Lamarca R, Ferrer M, Garcia-Aymerich J, Alonso J, Félez M (2002). Health-related quality of life and mortality in male patients with chronic obstructive pulmonary disease. Am J Respir Crit Care Med.

[25] Lumry WR, Castaldo AJ, Vernon MK, Blaustein MB, Wilson DA, Horn PT (2010). The humanistic burden of hereditary angioedema: impact on health-related quality of life, productivity, and depression. Allergy Asthma Proc.

[26] Bouillet L, Launay D, Fain O, Boccon-Gibod I, Laurent J, Martin L (2013). Hereditary angioedema with C1 inhibitor deficiency: clinical presentation and quality of life of 193 French patients. Ann Allergy Asthma Immunol.

[27] Gomide MA, Toledo E, Valle SOR, Campos RA, França AT, Gomez NP (2013). Hereditary angioedema: quality of life in Brazilian patients. Clinicas.

[28] Aabom A, Andersen K, Perez-Fernández E, Caballero T, Bygum A (2015). Health-related quality of life in Danish patients with hereditary angioedema. Acta Derm Venereol.

[29] Sánchez MD, Cuervo J, Rave D, Clemen G, Yepes JJ, Ortiz-Reyes B, et al. (2015) Hereditary angioedema in Medellín (Colombia): clinical evaluation and quality of life appraisal. 2015; 35:419–28 10.7705/biomedica.v35i3.2417 26849703

[30] Jindal NL, Harniman E, Prior N, Perez-Fernandez E, Caballero T, Betschel S (2017). Hereditary angioedema: health-related quality of life in Canadian patients as measured by the SF-36. Allergy Asthma Clin Immunol.

[31] Nordenfelt P, Nilsson M, Lindfors A, Wahlgren C-F, Björkander J (2017). Health-related quality of life in relation to disease activity in adults with hereditary angioedema in Sweden. Allergy Asthma Proc.

[32] Colás R, Muñoz P, Temprano R, Gómez C, Pascual J (2004). Chronic daily headache with analgesic overuse: epidemiology and impact on quality of life. Neurology.

[33] Targarona EM, Novell J, Vela S, Cerdán G, Bendahan G, Torrubia S (2004). Mid term analysis of safety and quality of life after the laparoscopic repair of paraesophageal hiatal hernia. Surg Endosc.

[34] Permanyer Miralda C, Brotons Cuixart C, Ribera Solé A, Moral Peláez I, Cascant Castelló P, Alonso J (2002). Resultados clínicos y de calidad de vida de los pacientes tratados con angioplastia coronaria con balón o stent. Estudio multicéntrico prospectivo Rev Española Cardiol.

[35] Bygum A, Andersen KE, Mikkelsen CS (2009). Self-administration of intravenous C1-inhibitor therapy for hereditary angioedema and associated quality of life benefits. Eur J Dermatol.

[36] Bewtra AK, Levy RJ, Jacobson KW, Wasserman RL, Machnig T, Craig TJ (2012). C1-inhibitor therapy for hereditary angioedema attacks: prospective patient assessments of health-related quality of life. Allergy Asthma Proc.

[37] Kreuz W, Martinez-Saguer I, Aygören-Pürsün E, Rusicke E, Heller C, Klingebiel T (2009). C1-inhibitor concentrate for individual replacement therapy in patients with severe hereditary angioedema refractory to danazol prophylaxis. Transfusion.

[38] Lumry WR, Miller DP, Newcomer S, Fitts D, Dayno J (2014). Quality of life in patients with hereditary angioedema receiving therapy for routine prevention of attacks. Allergy Asthma Proc.

[39] Rebollo P, Ortega F, Ortega T, Valdés C, García-Mendoza M, Gómez E (2003). Spanish validation of the & quot; kidney transplant questionnaire & quot; a useful instrument for assessing health related quality of life in kidney transplant patients. Health Qual Life Outcomes.

[40] Martínez Martín P, Frades B, Jiménez Jiménez FJ, Pondal M, López Lozano JJ, Vela L (1999). The PDQ-39 Spanish version: reliability and correlation with the short-form health survey (SF-36). Neurologia.

[41] Badia X, Díez-Pérez A, Alvarez-Sanz C, Díaz-López B, Diaz-Curiel M, Guillén F (2001). Measuring quality of life in women with vertebral fractures due to osteoporosis: a comparison of the OQLQ and QUALEFFO. Qual Life Res.

[42] Failde I, Ramos I (2000). Validity and reliability of the SF-36 Health Survey Questionnaire in patients with coronary artery disease. J Clin Epidemiol.

[43] Espinosa de los Monteros MJ, Alonso J, Ancochea J, González A (2002). Calidad de vida en asma: fiabilidad y validez del cuestionario genérico SF-36 aplicado a la población asmática de un área sanitaria. Arch Bronconeumol.

[44] Gómez-Besteiro MI, Santiago-Pérez MI, Alonso-Hernández Á, Valdés-Cañedo F, Rebollo-Álvarez P (2004). Validity and reliability of the SF-36 questionnaire in patients on the waiting list for a kidney transplant and transplant patients. Am J Nephrol.

[45] Squeglia V, Barbarino A, Bova M, Gravante C, Petraroli A, Spadaro G (2016). High attack frequency in patients with angioedema due to C1-inhibitor deficiency is a major determinant in switching to home therapy: a real-life observational study. Orphanet J Rare Dis.

[46] Zanichelli A, Azin GM, Cristina F, Vacchini R, Caballero T (2018). Safety, effectiveness, and impact on quality of life of self-administration with plasma-derived nanofiltered C1 inhibitor (Berinert®) in patients with hereditary angioedema: the SABHA study. Orphanet J Rare Dis.

[47] Lumry WR, Craig T, Zuraw B, Longhurst H, Baker J, Li HH (2018). Health-related quality of life with subcutaneous C1-inhibitor for prevention of attacks of hereditary angioedema. J Allergy Clin Immunol Pract.

[48] Ayuso-Mateos JL, Lasa L, Vázquez-Barquero JL, Oviedo A, Diez-Manrique JF (1999). Measuring health status in psychiatric community surveys: internal and external validity of the Spanish version of the SF-36. Acta Psychiatr Scand.

[49] Rentz A, Flood E, Altisent C, Bullinger M, Klamroth R, Garrido RP (2008). Cross-cultural development and psychometric evaluation of a patient-reported health-related quality of life questionnaire for adults with haemophilia. Haemophilia.

[50] Bunevicius A (2017). Reliability and validity of the SF-36 Health Survey Questionnaire in patients with brain tumors: a cross-sectional study. Health Qual Life Outcomes.

[51] Baiardini I, Bousquet PJ, Brzoza Z, Canonica GW, Compalati E, Fiocchi A (2010). Recommendations for assessing Patient-Reported Outcomes and Health-Related quality of life in clinical trials on allergy: a GA ^2^ LEN taskforce position paper. Allergy.

